# Atmospheric H_2_S exposure does not affect stomatal aperture in maize

**DOI:** 10.1007/s00425-020-03463-6

**Published:** 2020-09-24

**Authors:** Ties Ausma, Jeffrey Mulder, Thomas R. Polman, Casper J. van der Kooi, Luit J. De Kok

**Affiliations:** Laboratory of Plant Physiology, Groningen Institute for Evolutionary Life Sciences, University of Groningen, Groningen, The Netherlands

**Keywords:** Stomata, Transpiration, Signal molecule, Gasotransmitter, Sulfur metabolism, Air pollution

## Abstract

**Main conclusion:**

Stomatal aperture in maize is not affected by exposure to a subtoxic concentration of atmospheric H_2_S. At least in maize, H_2_S, thus, is not a gaseous signal molecule that controls stomatal aperture.

**Abstract:**

Sulfur is an indispensable element for the physiological functioning of plants with hydrogen sulfide (H_2_S) potentially acting as gasotransmitter in the regulation of stomatal aperture. It is often assumed that H_2_S is metabolized into cysteine to stimulate stomatal closure. To study the significance of H_2_S for the regulation of stomatal closure, maize was exposed to a subtoxic atmospheric H_2_S level in the presence or absence of a sulfate supply to the root. Similar to other plants, maize could use H_2_S as a sulfur source for growth. Whereas sulfate-deprived plants had a lower biomass than sulfate-sufficient plants, exposure to H_2_S alleviated this growth reduction. Shoot sulfate, glutathione, and cysteine levels were significantly higher in H_2_S-fumigated plants compared to non-fumigated plants. Nevertheless, this was not associated with changes in the leaf area, stomatal density, stomatal resistance, and transpiration rate of plants, meaning that H_2_S exposure did not affect the transpiration rate per stoma. Hence, it did not affect stomatal aperture, indicating that, at least in maize, H_2_S is not a gaseous signal molecule controlling this aperture.

**Electronic supplementary material:**

The online version of this article (10.1007/s00425-020-03463-6) contains supplementary material, which is available to authorized users.

## Introduction

Sulfur is an essential macronutrient for plants, which plants usually acquire as sulfate via the root (Hawkesford and De Kok 2006). After its uptake, sulfate is reduced via several intermediates to sulfide, which is subsequently incorporated in cysteine via the reaction of sulfide with *O*-acetylserine (OAS), catalyzed by the enzyme *O*-acetylserine(thiol)lyase (OAS-TL; Hawkesford and De Kok 2006). Cysteine functions as the precursor and reduced sulfur donor for the synthesis of other organic compounds.

It is often assumed that sulfur-containing metabolites might modulate physiological processes in plants. Hydrogen sulfide (H_2_S) might act as endogenous gasotransmitter that affects plant development and stress tolerance (Sirko and Gotor 2007; Calderwood and Kopriva 2014; Maniou et al. 2014; Hancock 2018). Moreover, H_2_S might control the aperture of stomata (Lisjak et al. 2010, 2011; Scuffi et al. 2014; Honda et al. 2015; Li et al. 2016; Aroca et al. 2018; Zhang et al. 2019). It is assumed that H_2_S is metabolized into cysteine to stimulate the synthesis of abscisic acid (ABA), which is the canonical trigger for stomatal closure (Batool et al. 2018; Rajab et al. 2019).

The physiological significance of H_2_S for stomatal closure should, however, be questioned. Research with thale cress (*Arabidopsis thaliana*), maize (*Zea mays*), cabbage (*Brassica olerecea*), pumpkin (*Curcubita pepo*), spruce (*Picea abies*), and spinach (*Spinacea oleracea*) showed that exposure to atmospheric H_2_S did not affect transpiration rates, measured at the whole plant level, at various concentrations and under all exposure periods applied (which ranged from minutes to days; De Kok et al. 1989; Van der Kooij and De Kok 1998; Stuiver and De Kok 2001; Tausz et al. 1998).

Accordingly, there are at least two caveats pertaining studies that reported impacts of H_2_S on stomatal dynamics. First, uncontrolled, potentially very high, levels of H_2_S have been used (e.g., Scuffi et al. 2014; Zhang et al. 2019). Sodium hydrosulfide (NaHS) has been used as H_2_S donor and it was added to nutrient or tissue incubation solutions at pH < 7.0. However, if NaHS is used at this pH range, HS^−^ is rapidly converted to gaseous H_2_S (HS^−^ + H^+^  ⇄ H_2_S; p*Ka* = 7.0; Lee et al. 2011). Since H_2_S is rather poorly soluble in water (the Henry’s law solubility constant for H_2_S is 0.086 M atm^−1^ at 25 °C), it is quickly released into the atmosphere, where it may transiently reach phytotoxic (growth-inhibiting) levels (Lee et al. 2011; Riahi and Rowley 2014). H_2_S may bind to metallo-groups in enzymes and other proteins (Beauchamp et al. 1984; Maas and De Kok 1988). Reported impacts of H_2_S on stomatal aperture could possibly be the consequence of such toxicity, instead of being specifically related to H_2_S functioning as gasotransmitter. One should further bear in mind that especially thale cress, which functioned as model plant, is rather susceptible to atmospheric H_2_S (Van der Kooij and De Kok 1998; Birke et al. 2015).

Secondly, in some studies (e.g., Zhang et al. 2019), mutants with a modified H_2_S homeostasis were used. Genetic manipulation of H_2_S homeostasis may not only alter tissue H_2_S content, but also the contents of other metabolites. These associated changes in metabolite contents may impact stomatal aperture. Hence, perceived impacts on stomatal aperture in mutants cannot directly be ascribed to the modification in H_2_S homeostasis (viz*.*, genotypic variation cannot directly be translated to phenotypic variation; Piersma and Van Gils 2011; Noble 2013; Noble et al. 2014).

The application of controlled, subtoxic (non-growth-inhibiting) levels of atmospheric H_2_S to non-mutant plants can provide a physiologically realistic view of the role of H_2_S in stomatal regulation. Plants absorb atmospheric H_2_S via stomata, since the leaf’s cuticle is hardly permeable for gases (Ausma and De Kok 2019). At the pH of leaf cells (i.e., ~ 5–6.4) absorbed H_2_S remains largely undissociated, causing it to easily pass cellular and subcellular membranes (Lee et al. 2011; Riahi and Rowley 2014). Foliar H_2_S levels increase significantly upon H_2_S fumigation (Ausma and De Kok 2019). For instance, exposure of thale cress to 0.5 and 1.0 µl l^−1^ H_2_S enhanced leaf H_2_S levels by approximately twofold and threefold, respectively (Birke et al. 2015). Since H_2_S is rapidly and with high affinity metabolized in cysteine, H_2_S fumigation also strongly enhanced foliar cysteine content and that of the tripeptide glutathione (De Kok et al. 1997; Birke et al. 2015; Ausma et al. 2017; Ausma and De Kok 2019). Thus, fumigation with low H_2_S levels may profoundly alter tissue sulfur status, without affecting plant growth (Ausma and De Kok 2019).

Plants may switch from using sulfate to using H_2_S as sulfur source: H_2_S absorbance by the foliage may partially downregulate the uptake and subsequent metabolism of sulfate (Buchner et al. 2004; De Kok et al. 1997). Plants may even grow with atmospheric H_2_S as the only sulfur source (viz*.,* in the absence of a root sulfate supply; De Kok et al. 1997; Koralewska et al. 2007, 2008). Whereas sulfate deprivation may reduce plant growth rate as well as endogenous cysteine and glutathione levels, fumigation with a sufficiently high H_2_S level may fully alleviate these reductions.

Here, we study the importance of H_2_S as gaseous signal molecule for the regulation of stomatal aperture in maize (*Zea mays*). Initially, we determined the H_2_S level that is subtoxic for maize, though sufficiently high to fully cover the plant’s sulfur demand for growth (viz*.*, the H_2_S concentration at which H_2_S-fumigated plants have a similar biomass as non-fumigated sulfate-sufficient plants). We then exposed plants for several days to this atmospheric H_2_S level in the presence or absence of a root sulfate supply. We measured plant growth, sulfur status, stomatal density, stomatal resistance, and transpiration rates. We conclude that, at least in maize, H_2_S is not a gaseous signal molecule that controls stomatal opening.

### Materials and methods

#### Plant material and growth conditions

Seeds of maize (*Zea mays*; cultivar number 669; Van Der Wal; Hoogeveen; The Netherlands) were germinated between moistened filter paper in the dark at 23 °C. After 3 days, the seedlings were put on 15 l boxes containing aerated tap water, which were placed in a climate-controlled room. Air temperature was 23 °C (± 1 °C), relative humidity was 60–70%, and the photoperiod was 16 h at a photon fluency rate of 300 ± 20 µmol m^–2^ s^–1^ (within the 400–700 nm range) at plant height, supplied by Philips GreenPower LED (deep red/white 120) production modules.

After 7 days, the seedlings were transferred to 13 l stainless-steel boxes (10 sets of plants per box, 6 plants per set in the first experiment, and 4 plants per set in the second experiment) holding aerated 50% Hoagland nutrient solutions, which were placed in 50 l cylindrical stainless-steel cabinets (0.6 m diameter) with a polymethyl-methacrylate top (Supplementary Fig. S1). Day and night air temperatures were 21 and 18 °C (± 1 °C), respectively, relative humidity was 30–40%, and the photoperiod was 16 h at a photon fluency rate of 300 ± 20 µmol m^–2^ s^–1^ (within the 400–700 nm range) at plant height, supplied by Philips GreenPower LED (deep red/white 120) production modules. Air exchange inside the cabinets was 40 l min^−1^ and the air inside the cabinets was stirred continuously by a ventilator. Nutrient solutions either contained 1 mM sulfate (+ S; sulfate-sufficient; solution’s composition being 2.5 mM CaCl_2_, 2.5 mM KCl, 0.5 mM KH_2_PO_4_, 1 mM MgSO_4_, 3.75 mM NH_4_NO_3_, 23.4 µM H_3_BO_3_, 4.8 µM MnCl_2_, 0.48 µM ZnSO_4_, 0.16 µM CuSO_4_, 0.26 µM Na_2_MoO_4_ and 45 µM Fe^3^^+^EDTA), or 0 mM sulfate (-S; sulfate-deprived; all sulfate salts replaced by chloride salts).

Plants were fumigated either with 0, 0.5, 1.0, or 1.5 µl l^−1^ H_2_S. Pressurized H_2_S diluted with N_2_ (1.0 ml l^−1^) was injected into the incoming air stream and the concentration in the cabinet was adjusted to the desired level using electronic mass flow controllers (ASM; Bilthoven; The Netherlands). H_2_S levels in the cabinets were monitored by an SO_2_ analyzer (model 9850) equipped with a H_2_S converter (model 8770; Monitor Labs; Measurements Controls Corporation; Englewood; CO; USA). Sealing of the lid of the boxes and plant sets prevented absorption of H_2_S by the nutrient solutions.

In the first experiment, plants were harvested after 10 days of exposure. In the second experiment after 7 days of exposure per treatment, sets of 4 plants were weighted (viz*.,* total biomass was determined). Subsequently, each plant set was transferred to a separate vessel containing 1.1 l of a similar 50% Hoagland nutrient solution as the set was grown on before (Supplementary Fig. S1). Vessels with plant sets were placed in the stainless-steel cabinets described above (with similar H_2_S levels) and plants were grown for an additional 3 days before harvest.

#### Growth analyses

Plant harvesting took place 3 h after the onset of the light period. To remove ions and other particles attached to the root, plants were placed with their roots in ice-cold de-mineralized water (3 × 20 s). Thereafter, the root and shoot were separated and weighted. In the second experiment, the shoot was additionally separated in leaf blades and the whorl of leaf sheaths (viz*.*, the seedlings did not yet possess a true stem, since all leaves emerged from the shoot base). Moreover, the total leaf blade area (abaxial plus adaxial) of the plants was determined by drawing the outlines of all leaf blades on graph paper.

#### Stomatal resistance

On the harvest day, stomatal resistance was analyzed at the abaxial and adaxial side of nascent leaf blades using a portable leaf porometer (AP4 Leaf Porometer; Delta-T-Devices Ltd.; Cambridge; UK). Measurements were performed 2–3 h after the onset of the light period.

#### Plant sulfur status

In whole shoots (leaf blades plus sheaths) and roots, which were stored at − 20 °C after harvest, sulfate levels were determined via high-performance liquid chromatography (HPLC) following Maas et al. (1986). Additionally, water-soluble non-protein thiols were extracted from freshly harvested shoots and roots. The total water-soluble non-protein thiol and cysteine content were determined colorimetrically according to De Kok et al. (1988).

#### Stomatal density

For the determination of stomatal density, silicone impression paste was prepared by 1:1 mixing of catalyst and base material (Provil Novo Light; Kulzer GmbH; Hanau; Germany). Subsequently, freshly harvested nascent leaf blades were gently pressed in the paste with either their abaxial or adaxial side. Once the paste had solidified, the leaf blades were removed and the mould was filled with transparent nail polish, as described by Kraaij and van der Kooi (2020). The positive (nail polish) replica was next examined under an Olympus CX-41 microscope and photographed using a Euromex CMEX 5000 camera with ImageFocus v3.0 software. From the obtained photographs, stomatal density (number of stomata per leaf area) was determined. Importantly, during trial experiments, also leaf sheaths were examined, but these did not hold stomata.

#### Transpiration rate

The transpiration rate of plants, expressed on a whole plant fresh weight basis, was calculated over the 3-day period that plants were grown on the vessels as follows:

1 $${I}_{\mathrm{t}} = \text{ }I\text{u }-I {\text{g}}$$

2 $${I}_{\mathrm{u}}=\left(\frac{(\ln{P}_{2}-\ln{P}_{1})}{3}\right){\cdot }\left(\frac{(I_{\text{m2}}-I_{\text{m1}}-8.95)}{{\text{(}P}_{2}-P_{1})}\right)$$

3 $${I}_{\mathrm{g}}=\left(\frac{(\ln{S}_{2}-\ln{S}_{1})}{3}\right){\cdot  0.9 +}\left(\frac{(\ln{R}_{2}-\ln{R}_{1})}{3}\right){\cdot  0.95}$$

where *I*_t_ represents the transpiration rate, *I*_u_ the water uptake rate, and *I*_g_ the amount of water required for plant growth (all expressed as g H_2_O g^−1^ FW plant day^−1^). Furthermore, *P* represents the whole plant’s fresh weight, *S* the shoot’s fresh weight, *R* the root’s fresh weight, and *I*_m_ the total solution weight in the vessels, with the subscripts 1 and 2 denoting the parameters’ value at the start and at the end of the 3-day exposure period, respectively. Moreover, whereas the factor 3 in the formulas refers to the 3-day duration of the experiment, the factor 8.95 refers to the average difference in solution weight of 4 vessels, which did not hold a plant set, between the start and end of the 3-day exposure period, respectively (standard deviation of this measurement was 0.61). Finally, the factors 0.9 and 0.95 represent the fraction of a maize shoot and root consisting of water, respectively (Ausma et al. 2017). It deserves mentioning that during the 3-day exposure period, the proportion of biomass allocated to the different plant organs was not affected.

#### Statistics

Statistical analyses were performed in GraphPad Prism (version 8.4.1; GraphPad Software; San Diego; CA; USA). Treatment means were compared using a two-way analysis of variance (ANOVA) with a Tukey’s HSD test as post hoc test at the *P* ≤ 0.05 level.

## Results and discussion

To test the relevance of H_2_S for the regulation of stomatal aperture, maize seedlings were grown with atmospheric H_2_S in the presence or absence of sulfate in the root environment.

We first assessed what H_2_S level is subtoxic for maize, albeit sufficiently high to fully cover the plant’s sulfur demand for growth. Sulfur-deficiency symptoms manifested after 10 days of sulfur deprivation (Table 1). The biomass of sulfate-deprived seedlings was on average 36% lower than that of sulfate-sufficient seedlings, which could be ascribed to both a lower root (33%) and shoot (37%) biomass (Table 1).

**Table 1:** Biomass of maize as affected by various levels of atmospheric H_2_S and sulfate deprivation. 10-day old maize was grown on a 50% Hoagland nutrient solution, containing 0 (-S) or 1.0 mM sulfate (+ S) and simultaneously fumigated with 0, 0.5, 1.0, and 1.5 µl l^−1^ H_2_S for 10 days. Data (g FW) represent the mean (± SD) of 5 measurements with 6 plants in each and different letters indicate significant differences between treatments (*P* ≤ 0.05; two-way ANOVA; Tukey’s HSD test as a post hoc test)

	0 µl l^−1^ H_2_S		0.5 µl l^−1^ H_2_S		1.0 µl l^−1^ H_2_S		1.5 µl l^−1^ H_2_S	
	+ S	− S	+ S	− S	+ S	-S	+ S	-S
Plant	3.60 ± 0.12a	2.31 ± 0.17b	3.78 ± 0.15a	3.66 ± 0.06a	3.71 ± 0.07a	3.63 ± 0.11a	1.74 ± 0.15c	1.77 ± 0.08c
Roots	1.30 ± 0.12a	0.87 ± 0.06b	1.39 ± 0.08a	1.33 ± 0.05a	1.36 ± 0.06a	1.33 ± 0.05a	0.96 ± 0.09b	0.96 ± 0.05b
Shoots	2.30 ± 0.08a	1.45 ± 0.17b	2.39 ± 0.11a	2.33 ± 0.04a	2.35 ± 0.11a	2.30 ± 0.08a	0.78 ± 0.08c	0.81 ± 0.04c

H_2_S fumigation can alleviate sulfur-deficiency symptoms. If maize was H_2_S fumigated in the absence of a sulfate supply, the plants did not develop any sulfur-deficiency symptoms (Table 1). The biomass of sulfate-deprived plants that were fumigated with 0.5 or 1.0 µl l^−1^ H_2_S was comparable to that of sulfate-sufficient, non-fumigated plants (Table 1), meaning that, analogous to the many plant species tested previously (Ausma et al. 2017; Ausma and De Kok 2019), maize can use H_2_S as a sulfur source. The results further demonstrate that maize is rather insusceptible for the potential phytotoxicity of H_2_S. Only exposure to 1.5 µl l^−1^ H_2_S negatively affected plant growth (Table 1). Generally, monocots are highly H_2_S tolerant (Stulen et al. 1990, 2000). In monocots, the shoot’s meristem is sheltered by the whorl of leaves. Therefore, H_2_S can hardly penetrate the meristem, which may explain why grasses are relatively H_2_S insusceptible (Stulen et al. 1990, 2000).

Tissue H_2_S, cysteine, and glutathione levels may be more profoundly affected at higher H_2_S levels (Birke et al. 2012; Ausma and De Kok 2019). Thus, in a second experiment, plants were fumigated with 1.0 µl l^−1^ H_2_S instead of 0.5 µl l^−1^ H_2_S. Similar to our previous observations (Table 1), sulfate-deprived plants had a lower biomass than sulfate-sufficient plants, owing to a lower root (34%) and leaf sheath biomass (22%; Table 2). Leaf blade biomass was comparable between sulfate-sufficient and sulfate-deprived plants (Table 2).

**Table 2:** Biomass of maize as affected by H_2_S fumigation and sulfate deprivation. 10-day old maize was grown on a 50% Hoagland nutrient solution, containing 0 (− S) or 1.0 mM sulfate (+ S) and simultaneously fumigated with 0 or 1.0 µl l^−1^ H_2_S for 10 days. Data (g FW) represent the mean (± SD) of 10 measurements with 4 plants in each and different letters indicate significant differences between treatments (*P* ≤ 0.05; two-way ANOVA; Tukey’s HSD test as a post hoc test)

	0 µl l^−1^ H_2_S		1.0 µl l^−1^ H_2_S	
	+ S	− S	+ S	− S
Plant	3.46 ± 0.11a	2.57 ± 0.10b	3.55 ± 0.23a	3.42 ± 0.19a
Roots	1.32 ± 0.11a	0.87 ± 0.08b	1.41 ± 0.14a	1.29 ± 0.14a
Leaf sheaths	1.90 ± 0.08a	1.49 ± 0.06b	1.92 ± 0.12a	1.90 ± 0.09a
Leaf blades	0.23 ± 0.01a	0.21 ± 0.02b	0.22 ± 0.01a	0.23 ± 0.02a

**Fig. 1:**
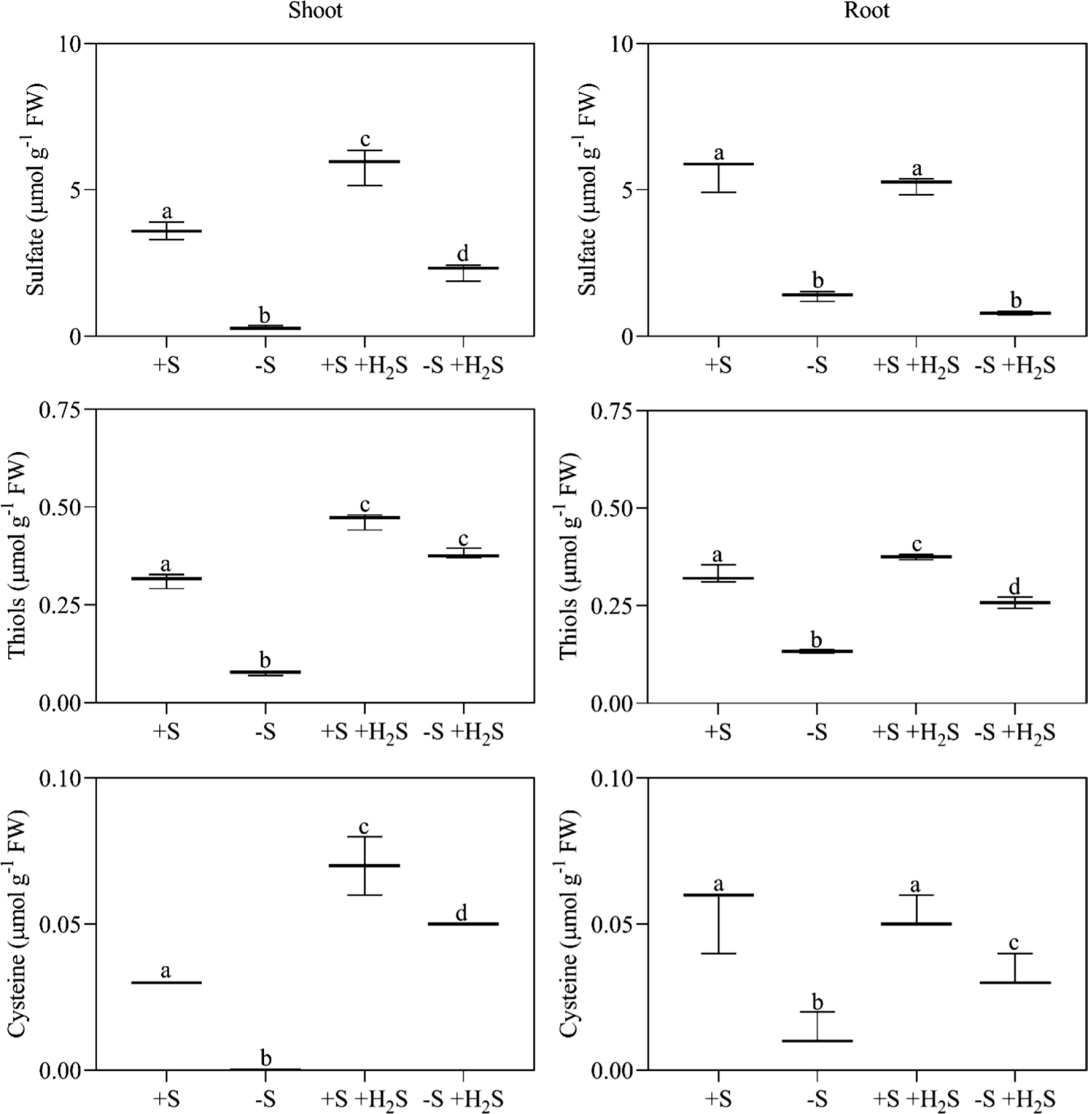
The content of sulfate, total water-soluble non-protein thiols, and cysteine in maize as affected by H_2_S fumigation and sulfate deprivation. For experimental details, see the legend of Table 2. Data, representing 3 measurements with 4 plants in each, are presented as boxes with a 5–95 percentile and whiskers. Different letters indicate significant differences between treatments (*P* ≤ 0.05; two-way ANOVA; Tukey’s HSD test as a post hoc test)

Sulfate deprivation lowered tissue sulfate and (water-soluble non-protein) thiol levels. Whereas a 10-day sulfate deprivation of maize reduced shoot and root sulfate levels by 92% and 75%, respectively, it reduced shoot and root thiol levels by 73% and 60%, respectively (Fig. 1). In plants, the thiol pool is mainly comprised of glutathione, though cysteine is a minor thiol (Buwalda et al. 1993). In maize, cysteine accounted for only 12% and 16% of the shoot and root thiol pool, respectively (Fig. 1). Sulfate deprivation decreased tissue cysteine contents: it lowered root and shoot cysteine content by 79% and 100%, respectively (Fig. 1). Clearly, the lower biomass production upon sulfate deprivation was accompanied by lower sulfate, glutathione, and cysteine contents (Fig. 1).

The biomass of plants that were fumigated with 1.0 µl l^−1^ H_2_S was comparable to that of sulfate-sufficient non-fumigated plants (Table 2). Thiol levels were higher in H_2_S-fumigated plants compared to non-fumigated plants (Fig. 1). Under sulfate-sufficient conditions, shoot total water-soluble non-protein thiol and cysteine levels were 1.4- and 2.0-fold higher in fumigated plants compared to non-fumigated plants, respectively (Fig. 1). Moreover, under sulfate-deprived conditions, fumigated plants had a 5.0-fold higher shoot total water-soluble non-protein thiol level, a 1.9-fold higher root water-soluble non-protein thiol level, and a 3.0-fold higher root cysteine level compared to non-fumigated plants (Fig. 1). Shoot cysteine levels in sulfate-deprived fumigated plants were even 1.5-fold higher compared to sulfate-sufficient non-fumigated plants (Fig. 1). Apparently, absorbed H_2_S was metabolized with high affinity into cysteine and subsequently into glutathione.

H_2_S-fumigated plants additionally had a higher shoot sulfate content compared to non-fumigated plants (Fig. 1). Whereas sulfate-sufficient fumigated plants had a 1.5-fold higher shoot sulfate content compared to sulfate-sufficient non-fumigated plants, sulfate-deprived fumigated plants had a 5.0-fold higher shoot sulfate content compared to sulfate-deprived non-fumigated plants (Fig. 1). The higher sulfate content in fumigated plants might be related to the oxidation of absorbed H_2_S and/or the degradation of excessively accumulated organic compounds (Ausma and De Kok 2019). However, it may also be due to H_2_S absorbance only partially downregulating root sulfate uptake (Ausma and De Kok 2019). Further research should elucidate the source of the accumulated sulfate.

**Fig. 2:**
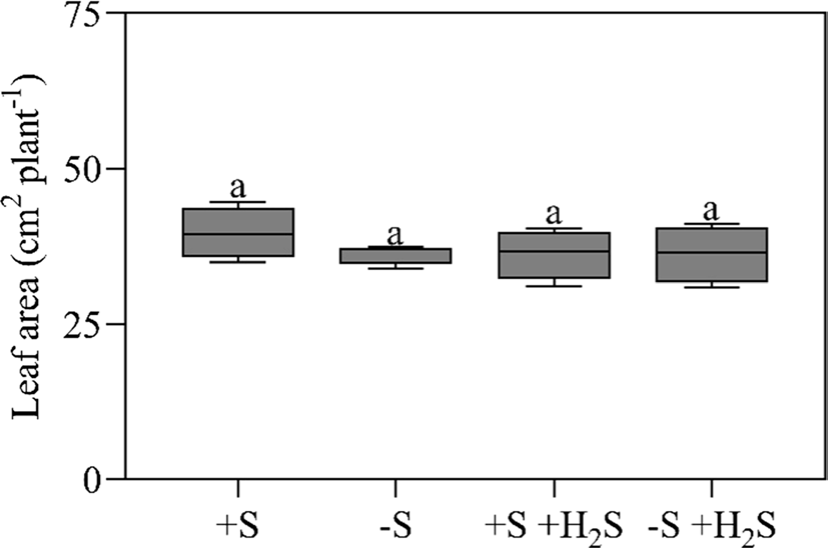
Total leaf blade area of maize as affected by H_2_S fumigation and sulfate deprivation. For experimental details, see the legend of Table 2. Data, representing 4 measurements with 4 plants in each, are presented as boxes with a 5–95 percentile and whiskers. Different letters indicate significant differences between treatments (*P* ≤ 0.05; two-way ANOVA; Tukey’s HSD test as a post hoc test)

**Fig. 3:**
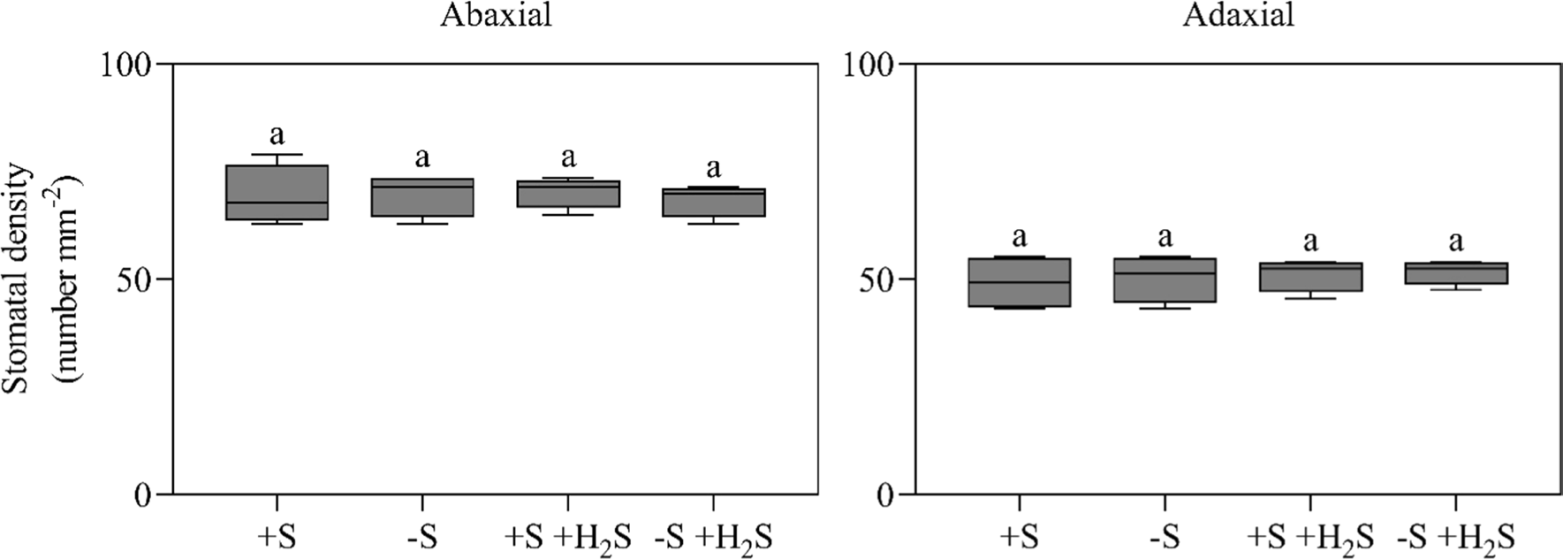
Stomatal density at the abaxial and adaxial side of leaf blades of maize as affected by H_2_S fumigation and sulfate deprivation. For experimental details, see the legend of Table 2. Data, representing 4 measurements with 2 plants in each, are presented as boxes with a 5–95 percentile and whiskers. Different letters indicate significant differences between treatments (*P* ≤ 0.05; two-way ANOVA; Tukey’s HSD test as a post hoc test)

Exposure of maize to 1.0 µl l^−1^ H_2_S did not affect the total leaf blade area and stomatal density at the abaxial and adaxial side of nascent leaves (Figs. 2 and 3). There were approximately 75 stomata mm^−2^ at the adaxial leaf side and 50 at the abaxial leaf side (Fig. 3). Similar densities were reported previously (e.g., Zheng et al. 2013). Based on these observations, it is concluded that H_2_S fumigation does not affect the total number of stomata per plant.

Based on these observations, it is also concluded that it is unlikely that H_2_S regulates the formation of aerenchyma in maize leaves. Aerenchyma can be formed via programmed cell death (PCD) events and H_2_S is hypothesized to be a signal molecule stimulating PCD (Maniou et al. 2014). However, H_2_S fumigation did neither alter leaf biomass nor leaf area (Figs. 2 and 3). It did thus not affect the specific leaf weight, which implies H_2_S did not induce aerenchyma formation in the foliage. In accordance with this result, previously, it was shown that exposure of maize to atmospheric H_2_S did not trigger the aerenchyma formation in roots (Ausma et al. 2017).

**Fig. 4:**
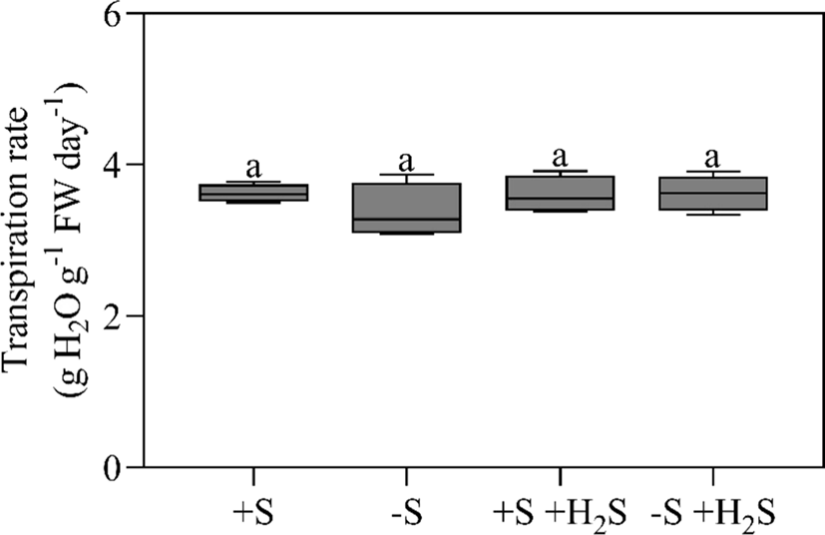
Transpiration rate of maize as affected by H_2_S fumigation and sulfate deprivation. For experimental details, see the legend of Table 2. Data, representing 4 measurements with 4 plants in each, are presented as boxes with a 5–95 percentile and whiskers. Different letters indicate significant differences between treatments (*P* ≤ 0.05; two-way ANOVA; Tukey’s HSD test as a post hoc test)

**Fig. 5:**
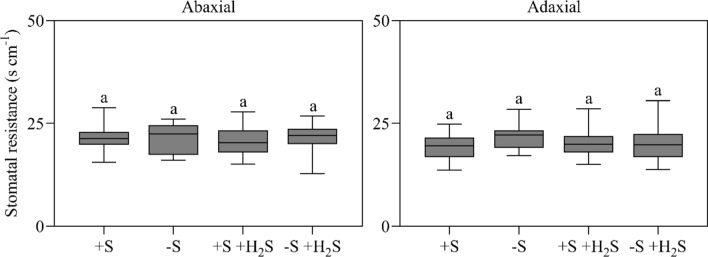
Stomatal resistance at the abaxial and adaxial side of leaf blades of maize as affected by H_2_S fumigation and sulfate deprivation. For experimental details, see the legend of Table 2. Data, representing 18 measurements on different plants, are presented as boxes with a 5–95 percentile and whiskers. Different letters indicate significant differences between treatments (*P* ≤ 0.05; two-way ANOVA; Tukey’s HSD test as a post hoc test)

Apart from having no effect on the total number of stomata per plant, exposure to 1.0 µl l^−1^ H_2_S did not affect the plants’ transpiration rate (Fig. 4). Transpiration rates were approximately 3.6 g H_2_O g^−1^ FW plant day^−1^ (Fig. 4). Accordingly, H_2_S exposure did not affect stomatal resistance at the abaxial and adaxial side of nascent leaves (Fig. 5). Since H_2_S fumigation did neither affect the total number of stomata per plant nor the plant’s transpiration rate and stomatal resistance, we conclude that fumigation did not affect the transpiration rate per stoma.

In maize and other plants, stomatal transpiration and conductance are strongly positively correlated with stomatal aperture (Shimshi 1963; Shimshi and Ephrat 1975; Lawson et al. 1998; Kaiser 2009). For instance, Shimshi (1963) reported for maize that stomatal conductance (*y*) depends on aperture (*x*) according to the formula *y* = 0.073 + 0.147*x* (*R*^2^ = 0.88). It thus is safe to say that fumigation with 1.0 µl l^−1^ H_2_S of maize did not modify stomatal aperture. The absence of an effect is not caused by H_2_S levels that are too low, because shoot cysteine levels were two-to-threefold higher in H_2_S-fumigated plants compared to non-fumigated plants (Fig. 1), which is highly similar to the twofold increase of foliar cysteine levels that Batool et al. (2018) reported to strongly impact stomatal aperture. Clearly, at least in maize, H_2_S does not interfere with the signal transduction cascade that regulates stomatal aperture.

## Conclusion

Maize plants could use atmospheric H_2_S as a sulfur source for growth. Foliar H_2_S absorbance markedly affected the plant’s sulfur status; however, it did not affect the total leaf area, stomatal density, stomatal resistance, and transpiration rate of plants. We thus conclude that, at least in maize, H_2_S does not function as signal molecule in the regulation of stomatal aperture.

### *Author contributions statement*

TA conceived and designed the study. TA, JM, and TRP collected the data. TA analyzed the data. TA, CJvdK, and LJDK wrote the manuscript.

## Electronic supplementary material

Below is the link to the electronic supplementary material.

Supplementary file1 (DOCX 2746 kb)
