# Supplementary material for: Atmospheric H2S exposure does not affect stomatal aperture in maize
Source: Planta. 2020 Sep 24;252(4):63. doi: 10.1007/s00425-020-03463-6 (PMC7511280; doi:10.1007/s00425-020-03463-6)

**Supplemental Fig. S1** The experimental set-up used in the described research. Plants were grown in cylindrical stainless-steel cabinets on either 13 l boxes (left photo) or 1.1 l vessels (right photo), holding 50% aerated Hoagland nutrient solutions. For additional experimental details, see the main text.
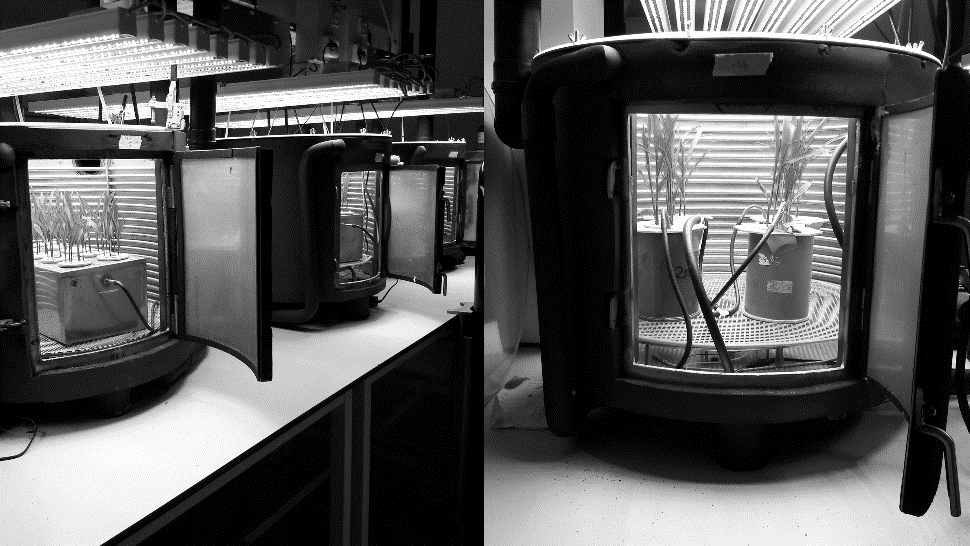

Supplement: Supplementary file 1 — Supplementary file1 (DOCX 2746 kb) [file 425_2020_3463_MOESM1_ESM.docx]
